# The Effect of Different Thawing Rates on Cryopreserved Human Iliac Arteries Allograft's Structural Damage and Mechanical Properties

**DOI:** 10.1155/2020/6545190

**Published:** 2020-10-08

**Authors:** Robert Novotny, Pavel Mericka, Jaroslav Chlupac, Roman Matejka, Jakub Kristek, Tomas Marada, Miroslav Konarik, Peter Ivak, Lubomir Sterba, Jaroslav Hlubocky, Jan Pirk, Libor Janousek, Jiri Fronek

**Affiliations:** ^1^Transplant Surgery Department, Institute for Clinical and Experimental Medicine, Prague, Czech Republic; ^2^Tissue Bank, Faculty Hospital Hradec Kralove, Hradec Kralove, Czech Republic; ^3^Department of Anatomy, Second Faculty of Medicine, Charles University in Prague, Czech Republic; ^4^Department of Biomedical Engineering, Faculty of Biomedical Engineering, Czech Technical University, Prague, Czech Republic; ^5^Department of Cardiovascular Surgery, Institute for Clinical and Experimental Medicine, Prague, Czech Republic; ^6^2nd Department of Cardiovascular Surgery, General University Hospital, Prague, Czech Republic

## Abstract

**Introduction:**

The rate of thawing of cryopreserved human iliac arteries allografts (CHIAA) directly affects the severeness of structural changes that occur during this process.

**Method:**

The experiment was performed on ten CHIAA. The 10% dimethylsulphoxide in 6% hydroxyethyl starch solution was used as the cryoprotectant; all CHIAA were cooled at a controlled rate and stored in the vapor phase of liquid nitrogen (-194°C). Two thawing protocols were tested: (1) placing the CHIAA in a water bath at 37°C, and (2) the CHIAA were thawed in a controlled environment at 5°C. All samples underwent analysis under a scanning electron microscope. Testing of the mechanical properties of the CHIAA was evaluated on a custom-built single axis strain testing machine. Longitudinal and circumferential samples were prepared from each tested CHIAA.

**Results:**

Ultrastructural analysis revealed that all five CHIAA thawed during the thawing protocol 1 which showed significantly more damage to the subendothelial structures when compared to the samples thawed in protocol 2. Mechanical properties: Thawing protocol 1—longitudinal UTS 2, 53 ± 0, 47 MPa at relative strain 1, 27 ± 0, 12 and circumferential UTS 1, 94 ± 0, 27 MPa at relative strain 1, 33 ± 0, 09. Thawing protocol 2—longitudinal ultimate tensile strain (UTS) 2, 42 ± 0, 34 MPa at relative strain 1, 32 ± 0, 09 and circumferential UTS 1, 98 ± 0, 26 MPa at relative strain 1, 29 ± 0, 07. Comparing UTS showed no statistical difference between thawing methods.

**Conclusion:**

Despite the significant differences in structural changes of presented thawing protocols, the ultimate tensile strain showed no statistical difference between thawing methods.

## 1. Introduction

Vascular infection is a leading cause of morbidity and mortality affecting up to 15% of patients [1]. Cryopreserved arterial allografts (CA) are used as an alternative vascular conduit in the treatment of vascular infection or critical limb ischemia in patients with the absence of autologous saphenous vein, or patients unsuitable for prosthetic graft implantation due to a high risk of reoccurring infection [2]. Despite the need for CA frequent reintervention and poor amputation-free survival, CA are used as the last option in patients where fresh allografts are unavailable [3]. Gilbert et al. published in 1979 the first concept on preserving mechanical properties of CA [4]. In the past two decades, there were significant technical improvements and innovations in tissue cryopreservation minimising the structural damage and preserving functional integrity of vascular allografts [5]. However, minimal progress in thawing protocols of the CA abated the progress in cryopreserving techniques incontrovertibly causing structural damage of CA. Therefore, it is essential to evaluate these structural changes caused by thawing and their effect on CA mechanical properties. To our knowledge, the correlation between structural changes caused by different rates of thawing of CA and their subsequent impact on the mechanical properties has not been reported on human CA.

All the allografts were harvested in the operation theatre in patients that were organ donors and were pronounced “clinically dead” with compliance to the Czech Republic's transplants laws. All experiments were performed in accordance with relevant guidelines and regulations of the Czech Republic. All departments (Transplant Surgery Department, Institute for Clinical and Experimental Medicine, Prague, Czech Republic; Tissue Bank, Faculty Hospital Hradec Kralove, Hradec Kralove, Czech Republic) have approved regulations dealing with experimental work on cryopreserved human tissues. Individual consents for the use of tissue are not available as the allografts are not stored under the name of the donor, and the individual donor cannot be traced. The experimental work was performed only on allografts that were removed from the tissue bank as “unsuitable” for patient transplant.

This study was reviewed and approved by the Ethical Committee of Faculty Hospital Hradec Kralove, Hradec Kralove, Czech Republic, without the need for inform consent.

## 2. Materials and Methods

### 2.1. Sample Preparation

#### 2.1.1. Allograft Characteristics

Basic allograft characteristics: thawing protocol 1(thawing in a water bath at +37°C): 4 (80%) males, average age: 46.8 years. Thawing protocol 2 (thawing in a controlled environment at 5°C): 5 (100%) males, average age: 39.6 years (Table 1).

#### 2.1.2. Allograft Harvest, Processing, and Cryopreservation Protocol

All human CHIAA were harvested aseptically in deceased donors during multiple organ/tissue harvests performed at operating rooms of organ donor hospitals. Organ preservation solution Celsior (IGL, France) supplemented with gentamycin was used for transport to a tissue bank included in the list of European Union Tissue Establishments under the EUTE Code CZ000427. The grafts were processed as soon as possible after admission and/or input control [2]. Decontamination was performed in an antibiotic cocktail described by van Kats at 37°C for 2 hours [6]. The remnants of antibiotics were washed out by placing the allografts into the 6% hydroxyethyl starch (HES) solution for 20 minutes. Placement of the allografts to the precooled cryoprotectant solution 10% dimethylsulphoxide (DMSO) (WAK Chemie, GMBh, FRG) in 6% HES (Fresenius–Kabi, FRG)) was performed in the cleanrooms of the tissue bank (cleanroom of the grade A with the background of the grade B) and packed by the double-layer technique (Eva bags, Maco-Biotech, France). After the packaging was completed, the bag was placed into a metal cassette and cooled at a controlled rate of -1°C/min from +17°C to -90°C, 5 K/min till -150°C in a programmable freezer Kryo 10/16 (Planer Biomed, UK). Stored in a vapor phase of liquid nitrogen in a container Kryo-CE 10 K (Taylor –Wharton, USA) equipped with automatic filling, and continuous registration of the temperature and liquid nitrogen levels at -194°C [7]. The grafts were thawed after the 1- to 4-year period of storage.

#### 2.1.3. Thawing Protocols

Experimental work was based on investigating 10 CHIAA. CHIAA were randomly divided into two groups of five samples. All CHIAA were thawed in their unopened original packaging. Two thawing protocols were tested: protocol 1: five CHIAA thawed in a water bath at 37°C. Thawing times: minimum 3 min. 17 sec. and maximum 3 min. 30 sec. Protocol 2: five CHIAA thawed in a controlled environment at 5°C. Thawing times: minimum of 90 min and maximum of 90 min.

The identical times in both thawing protocols were given by the standardised amount of cryoprotectant used, similar length and thickness of the CHIAA. After the thawing was completed, two samples were taken from each CHIAA. The first sample was fixed in a 4% formaldehyde solution and sent for scanning electron microscope (SEM) analysis. The second sample was stored in a Celsior solution (batch CEL 0076-02) and sent for immediate testing of the mechanical properties.

### 2.2. Sample Preparation for Ultrastructural Analysis

A 2 cm sample from each CHIAA from both thawing protocols was divided into 5-10 mm subsamples. Samples were mounted on convex polystyrene casts. All samples were washed in distilled water for 5 min, then dehydrated in a graded ethanol series (70, 85, 95, and 100%) for 5 min at each level. The tissue samples were then immersed in 100% hexamethyldisilane (CAS No. 999-97-3; Fluka Chemie AG, Buchs, Switzerland) (HMDS) for 10 minutes and air-dried in an exhaust hood at room temperature.

Processed samples were mounted on stainless steel stubs, coated with gold, and stored in a desiccator until they were studied and photographed by electron microscope on scanning mode operating at 25 kV–BS 301. A modified scoring system designed by Krs et al. was used for analyses of the morphological changes of the CHIAA under the electron microscope (Table 2) [8].

### 2.3. Testing of the Mechanical Properties

After the thawing protocols were completed, a sample from each CHIAA was collected and stored in a Custodiol® solution. Afterwards, they were sent for immediate testing of the mechanical properties. Longitudinal and circumferential samples were prepared from each tested CHIAA. These tissues were cut to stripes approximately 3 × 30 mm. Exact dimensions of individual samples were measured using a calibrated microscope camera to obtain the crossectional area and sample initial length. Testing of the mechanical properties of the CHIAA was evaluated on a custom-built uniaxial tensile testing machine. This machine consists of a servo-controlled linear stage with 3D printed tissue fixing jaws with strain sensors. Controlling was done using the NI compact DAQ (National Instruments, Austin, TX, USA) platform. The linear stage was controlled using the digital I/O module (cDAQ 9401), and a signal from the strain sensor was acquired using a universal analogue module with signal conditioning (cDAQ 9218). Custom LabVIEW software was used. Servo control was synchronized with the data acquisition sample rate. The samples' stress-strain data were measured until rupture. The ultimate tensile stress and the relative strain were evaluated.

## 3. Results

**Figure 1:**
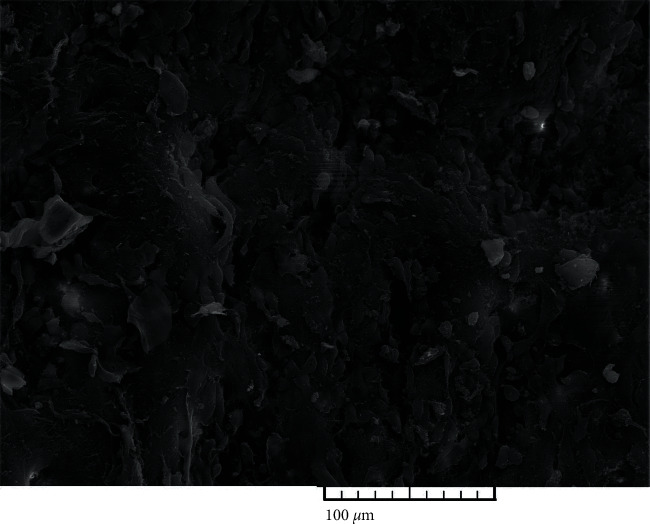
Thawing protocol 1 (thawing at water bath at 37°C)—cryopreserved human iliac artery allograft (CHIAA) (magnification: 500x). CHIAA: complete loss of endothelium, dispersion of defects or fractures in subendotlium (arrow), and very gentle longitudinal corrugations parallel to the direction of blood flow.

**Figure 2:**
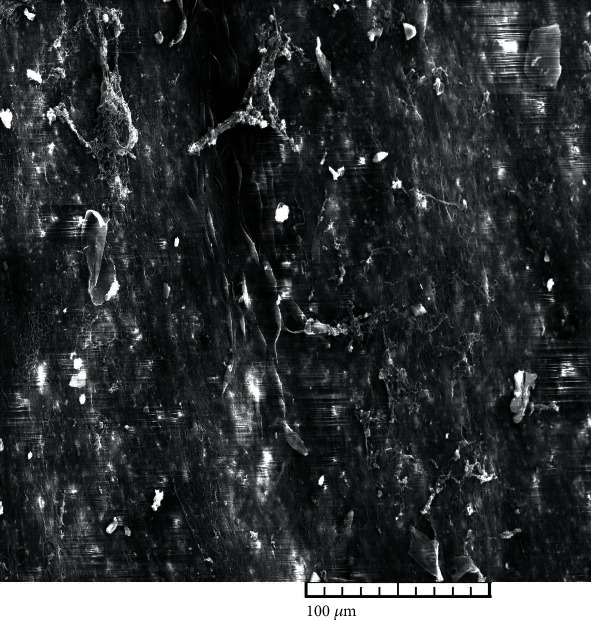
Thawing protocol (thawing in a controlled environment at 5°C)—cryopreserved human iliac artery allograft (CHIAA) (magnification: 500x). CHIAA: complete loss of endothelium, no contraction of smooth muscle cells, no dispersion of defects or fractures in subendotlium, and basal lamina exposed with no damage.

The ultrastructural analysis of the CHIAA was as follows: thawing protocol 1(thawing in a water bath at 37°C): 5 (100%) complete loss of endothelium, 5 (100%) gentle smooth muscle cells contraction, 5 (100%) dispersion of defects or fractures in subendothelium, 5 (100%) very gentle longitudinal corrugations parallel to the direction of blood flow, and 5 (100%) basal lamina damage (Figure 1). Scoring by the Krs et al. system (Error! Bookmark not defined.): 5 (100%) score, 6—damage of subendothelial layers. Thawing protocol 2 (thawing in a controlled environment at 5°C): 5 (100%) complete loss of endothelium, 5 (100%) no contraction of smooth muscle cells, 5 (100%) no dispersion of defects or fractures in subendothelium, and 5 (100%) basal lamina exposed with no damage (Figure 2). Scoring by the Krs et al. system (Error! Bookmark not defined.): 5 (100%) score, 5—complete loss of endothelium.

**Figure 3:**
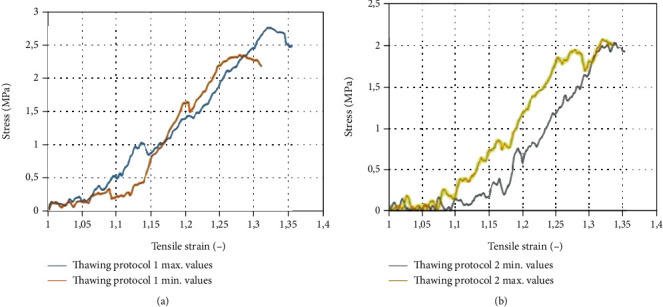
Mechanical properties of cryopreserved human iliac arteries allografts: longitudinal direction. (a) Thawing protocol 1 (thawing in a water bath at 37°C). (b) Thawing protocol 2 (thawing in a controlled environment at 5°C).

**Figure 4:**
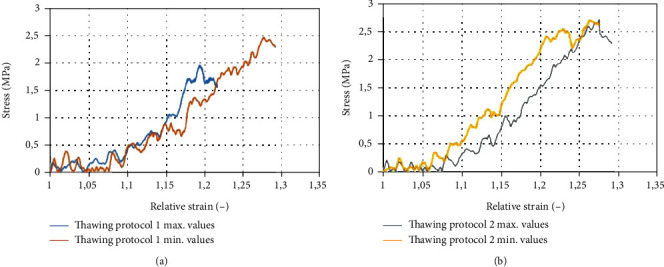
Mechanical properties of cryopreserved human iliac arteries allografts: circumferential direction. (a) Thawing protocol 1 (thawing in a water bath at 37°C). (b) Thawing protocol 2 (thawing in a controlled environment at 5°C).

The mechanical properties of the CHIAA were as follows: thawing protocol 1 (thawing in a water bath at 37°C)—longitudinal ultimate tensile strain (UTS) 2, 53 ± 0, 47 MPa at relative strain 1, 27 ± 0, 12 and circumferential UTS 1, 94 ± 0, 27 MPa at relative strain 1, 33 ± 0, 09 (Figure 3). Thawing protocol 2 (thawing in a controlled environment at 5°C)—longitudinal UTS 2, 42 ± 0, 34 MPa at relative strain 1.32 ± 0.09 and circumferential UTS 1, 98 ± 0, 26 MPa at relative strain 1, 29 ± 0, 07 (Figure 4). Comparing UTS showed no statistical difference between thawing methods.

## 4. Discussion

A prototypical CA should have anatomical and physiological properties indistinguishable from a native artery. The viscoelastic and inertial properties of the arterial wall are essential for proper arterial functional. They allow arteries to convert blood pulsatility into continuous arteriolar-capillary flow and pressure [9]. Whether these properties are affected by cryopreservation and subsequent thawing remain controversial [10].

Experimental models dealing with CA functional properties and structural damage caused by cryopreservation have been published scarcely [11]. Nevertheless, research papers dealing with similar experimental models on human CA are even rarer [12–14]. However, none of these papers had tried to correlate structural changes of human CA caused by thawing and their direct effect on CA mechanical properties. Our experimental work tried to find the correlation between the severity of structural damage of CA that occurs during thawing and mechanical properties. Cryopreservation of vascular allografts is done in a manner that minimises the structural damage, preserves structural integrity, and aims to maintain hemodynamic properties of a native vessel [15]. Karls-son et al. and Rigol et al. described damages caused by cryopreservation on the smooth muscle cells, elastin, and collagen fibres [16, 17]. Several papers showed that after cryopreservation of arteries affects their reactivity, the endothelial integrity and function are impaired [9, 18]. Our experimental work showed that the severity of structural damage could be correlated to the rate of thawing. CA thawed at slower thawing protocol showed much lower structural damage when compared to CA thawed at a higher rate.

A crucial concern in the use of CA is their potential dilatation, aneurysm formation, and eventual rupture caused by structural damage which caused during thawing. Furthermore, this can alter the CA viscoelastic and inertial properties. Shahmansouri et al. demonstrated that fresh allografts (FA) showed 20% higher toughness than CA, demonstrating the effect of cryopreservation on mechanical properties, thus adversely affecting the toughness properties of the CA [15]. Their work showed that the variations in wall damping could cause changes in the strain and stress in the arterial wall [15]. Kubikova et al. showed the wave-like organisation of collagen in arteries, contributing to tissue mechanical properties only at higher values of the deformation after its alignment in the loading direction. The initial phase of loading the material properties of collagen only marginally influences the modulus of elasticity of tissue [19]. Other aspects that may increase the risk of rupture by creating increased localised wall stress are arterial calcifications [19]. However, intimal calcifications are regularly found in the context of an atherosclerotic alteration of the vessel wall. Their role in an increased risk of rupture is not well understood. Our experimental results showed that the biomechanical properties of CA were not significantly affected by the rate of thawing. Although, due to our small sample size, we cannot draw a strong conclusion, and more testing is required.

The biggest limitation of our experimental work is the small number of CHIAA used for ultrahistological analysis. Also, the technical aspect of the mechanical property testing of the CHIAA did not allowed us to provide the modulus elastic values.

## 5. Conclusion

Human arterial allografts are very rarely available for the experimental work. The cryopreservation protocol used by our tissue bank preserves the biomechanical properties of vascular allografts despite the rate of thawing. Slow thawing of vascular allografts greatly reduces the structural damage caused by cryopreservation and subsequent thawing. Despite the significant differences in structural changes of presented two thawing protocols, the ultimate tensile strain showed no statistical difference between thawing methods. This does not mean that the structural changes occurring during fast thawing do not affect the clinical performance of cryopreserved arterial allografts in other aspects.

## Figures and Tables

**Table:** (a) Thawing protocol 1 (thawing in a water bath at +37°C)

Gender	Donor age	ABO, RH compatibility	Date of cryopreservation
Male	55	0+	30.01.2016
Male	42	A+	08.08.2015
Female	26	0+	23.04.2014
Male	56	A+	31.07.2017
Male	55	0+	30.01.2016

**Table:** (b) Thawing protocol 2 (thawing in a controlled environment at 5°C)

Gender	Donor age	ABO, RH compatibility	Date of cryopreservation
Male	22	AB+	23.04.2014
Male	45	AB+	08.08.2015
Male	22	AB+	23.04.2014
Male	49	AB+	31.07.2017
Male	60	A+	30.01.2016

**Table 2:** Scoring system used for the morphological analysis of the cryopreserved human iliac arteries allograft.

Score	Morphology
1	Morphologically intact endothelium—putative physiological changes are not reflected in the superficial morphology of endothelial cells
2	Confluent endothelium with structural inhomogeneity—irregularities in the form of individual cells and changes of their membranes are detectable
3	Disruption of intercellular contacts—continuity of endothelial coverage is lost, and endotheliocytes shrink while still adhering to basal membrane
4	Separation of endothelial cells—endotheliocytes separated from the basal lamina. Initially, they protrude by their intercellular edges into the lumen
5	Complete loss of endothelium—denudation of the endothelial covering with the basal lamina exposed
6	Damage of subendothelial layers—the luminal surface is covered only by remnants of basal membrane, and the fiber structure of the lamina fibrosa and the lamina ventricularis may be dissolved

## Data Availability

All of the data is presented within the manuscript.
